# Vornorexant versus lemborexant for insomnia disorder: a systematic review and network meta-analysis

**DOI:** 10.1093/ijnp/pyag047

**Published:** 2026-09-01

**Authors:** Taro Kishi, Kenji Sakuma, Tsuyoshi Kitajima, Nakao Iwata

**Affiliations:** Department of Psychiatry, Fujita Health University School of Medicine, Toyoake, Aichi 470-1192, Japan; Department of Psychiatry, Fujita Health University School of Medicine, Toyoake, Aichi 470-1192, Japan; Department of Psychiatry, Fujita Health University School of Medicine, Toyoake, Aichi 470-1192, Japan; Department of Psychiatry, Fujita Health University School of Medicine, Toyoake, Aichi 470-1192, Japan

**Keywords:** vornorexant, lemborexant, network meta-analysis, sleep initiation and maintenance disorders, insomnia disorder

## Abstract

**Objective:**

This frequentist network meta-analysis was conducted to compare vornorexant (VOR) and lemborexant (LEM) in terms of efficacy, tolerability, and safety outcomes in adults with insomnia disorder.

**Methods:**

Efficacy outcomes evaluated at weeks 1 and 2 included subjective time to sleep onset (sTSO), which served as the primary outcome at week 2, as well as subjective total sleep time (sTST), subjective wake after sleep onset (sWASO), and subjective sleep efficiency. Tolerability outcome was treatment discontinuation due to adverse events. Safety outcomes included the incidence of death, suicidal behavior or ideation, at least one adverse event, somnolence, fatigue, dizziness, falls, headache, nightmares, cataplexy, and sleep paralysis.

**Results:**

This network meta-analysis included seven trials (*n* = 2805, average age = 55.14 years, 69.22% female). Treatment arms comprised VOR 5 mg/day (VOR5), VOR 10 mg/day (VOR10), LEM 5 mg/day (LEM5), LEM 10 mg/day (LEM10), and placebo. All active treatments were associated with significantly greater improvements in sTSO at week 2 than placebo. The mean differences in sTSO at week 2 were −11.997 min (95% CI, −15.236 to −8.757) for LEM5, −14.856 min (95% CI, −18.038 to −11.674) for LEM10, −11.364 min (95% CI, −14.733 to −7.996) for VOR5, and − 9.758 min (95% CI, −13.186 to −6.330) for VOR10. LEM5, LEM10, and VOR5 demonstrated significant improvements across all efficacy outcomes at weeks 1 and 2 over placebo. However, although VOR10 did not demonstrate superiority over placebo for sWASO at weeks 1 and 2, it was superior to placebo for all other efficacy outcomes. LEM5 and LEM10 were associated with a higher incidence of at least one adverse event and somnolence compared with placebo, whereas no significant differences in tolerability or safety outcomes were observed for VOR5 or VOR10 versus placebo.

**Conclusions:**

Dosage variations could influence both the clinical efficacy and safety profile of these agents.

**Significant outcomes**

- All active treatment groups (LEM5, LEM10, VOR5, and VOR10) demonstrated significantly greater improvements in sTSO at week 2 compared with placebo. Among the active treatments, LEM10 consistently demonstrated the greatest effect size across all efficacy outcomes.
- A dose-dependent increase in effect size was observed for LEM when comparing the 5 mg and 10 mg doses; in contrast, no comparable dose–response pattern was evident for VOR, as the 10 mg dose failed to demonstrate statistical superiority over placebo for sWASO.
- Both LEM5 and LEM10 were associated with a significantly higher incidence of at least one adverse event and somnolence than placebo. Conversely, VOR5 and VOR10 demonstrated favorable safety and tolerability profiles, showing no significant differences in adverse outcomes versus placebo.

**Limitations**

- The meta-analysis included a small number of studies and participants.
- The observation period was limited to 2 weeks, consistent with the study duration of the VOR trial. All comparisons between VOR and LEM were based on indirect evidence from a network meta-analysis.

## Introduction

Vornorexant (VOR), a dual orexin receptor antagonist (DORA), was introduced in 2025 for the treatment of insomnia.^1^ It is rapidly absorbed and has a terminal half-life (t₁/₂) of approximately 2 h, making it shorter acting than other DORAs, including suvorexant, lemborexant (LEM), and daridorexant, while remaining comparable to zolpidem.^1^ Based on these pharmacokinetic properties, VOR is expected to be particularly effective in adults with difficulty in initiating sleep. Notably, VOR is the only hypnotic agent approved in Japan without restrictions on vehicle operation.^2^ However, this regulatory status does not indicate a complete absence of next-day impairment in all patients. Therefore, individual susceptibility to potential residual effects should remain a critical clinical consideration.

In our previous network meta-analysis, LEM showed a larger effect size in improving difficulty in initiating sleep than suvorexant and daridorexant.^3^^,^^4^ However, clinically meaningful differences in terms of efficacy, tolerability, and safety between VOR and LEM in adults with insomnia disorder remain uncertain because no randomized controlled trials (RCTs) have directly compared these agents in this population. Network meta-analysis enables indirect comparisons by integrating evidence from RCTs comparing VOR with placebo and LEM with placebo.^5^ Therefore, we conducted a network meta-analysis to compare the efficacy, tolerability, and safety of VOR and LEM with insomnia disorder.

## Materials and methods

This study adhered to the Preferred Reporting Items for Systematic Reviews and Meta-Analyses guidelines^6^ (Table S1) and Cochrane Handbook for Systematic Reviews of Interventions.^5^ The protocol was registered with the Open Science Framework (https://osf.io/u9nhe/overview). At least two authors independently validated the literature search, data transfer accuracy, and data calculations. This was a systematic review and meta-analysis of previously published data. Therefore, institutional review board approval and informed consent were not required.

### Search strategy and inclusion criteria

The “*PICOS*” strategy was used for systematic literature review as follows: *Participants* were adults with insomnia disorder; *Intervention* included VOR and/or LEM; *Control* was a placebo; *Outcomes* included efficacy, tolerability, and safety; and *Studies* were double-blind, randomized, placebo-controlled trials. The inclusion criteria were as follows: (1) studies lasting at least 2 weeks that included VOR or LEM; (2) studies enrolling adult patients with insomnia diagnosed according to any recognized diagnostic criteria. The exclusion criteria were as follows: (1) studies enrolling pediatric or adolescent participants; (2) studies evaluating only dose regimens not recommended in the United States,^7^ Europe,^8^ or Japan.^2^ Discrepancies in article selection were resolved by consensus among the authors. Multiple publications or conference abstracts reporting the same study were identified by verifying the clinical trial registration number and/or referencing previous review articles.

### Outcome measures, data synthesis, and data extraction

The primary outcome was subjective time to sleep onset (sTSO) at week 2. Other efficacy outcomes evaluated at weeks 1 and 2 included sTSO, subjective total sleep time (sTST), subjective wake after sleep onset (sWASO), and subjective sleep efficiency. Tolerability outcomes included treatment discontinuation due to adverse events. Safety outcomes comprised the incidence of death, suicidal behavior or ideation, at least one adverse event, somnolence, fatigue, dizziness, falls, headache, nightmares, cataplexy, and sleep paralysis.

This network meta-analysis included outcomes that were reported in both the VOR and LEM trials. Data were extracted from the intention-to-treat or modified intention-to-treat populations. Complete case analyses were not used.

### Meta-analysis methods

A frequentist network meta-analysis with a random-effects model was conducted to estimate mean differences and odds ratios, with corresponding 95% confidence intervals (CIs).^9^^,^^10^ Network heterogeneity was evaluated using *τ*^2^ statistics. Statistical inconsistency was assessed using the design-by-treatment interaction test for global inconsistency and Separating Indirect from Direct Evidence test for local inconsistency. Surface under the curve cumulative ranking (SUCRA) probabilities were utilized to rank treatments for each outcome. Two VOR trials had a treatment duration of 2 weeks.^11^^,^^12^ To satisfy the transitivity assumption of the network, all efficacy data were extracted from LEM trials^13–17^ at weeks 1 and 2 including data for other outcomes from LEM trials at week 2.^18^ The sufficiency of the distribution differences was evaluated to validate the analysis by comparing the distribution of possible effect modifiers across included treatments in the network meta-analysis using the Kruskal–Wallis test for continuous variables and Pearson chi-square test or Fisher’s exact test for categorical variables. The effects of these potential modifiers on treatment outcomes were further explored using network meta-regression analyses. Potential confounding factors included the proportion of female participants, mean age, proportion of participants aged *>*65 years, and total sample size (Table S2). Data were insufficient to perform a network meta-analysis of death, suicidal behavior and ideation, fatigue, dizziness, falls, nightmares, cataplexy, and sleep paralysis. Therefore, given the clinical importance of these adverse events, their incidence was estimated using single-group meta-analyses. The overall risk of bias for each trial was assessed using the Cochrane risk of bias tool for randomized trials version 2 (https://www.riskofbias.info/). Finally, the results were integrated into the Confidence in Network Meta-Analysis application—an adaptation of the Grading of Recommendations Assessment, Development, and Evaluation approach—to validate the credibility of each network meta-analysis.

## Results

Figure S1 presents the literature search flowchart and provides a detailed description of the study selection process. Of the 73 articles identified, 35 were duplicates. After screening titles and abstracts, 36 articles were excluded. The previous reviews and medication package insert identified five additional studies.^2–4^ Finally, the meta-analysis included seven trials (*n* = 2805, average age = 55.14 years, 69.22% female).^11–17^ Treatment arms comprised VOR 5 mg/day (VOR5), VOR 10 mg/day (VOR10), LEM 5 mg/day (LEM5), LEM 10 mg/day (LEM10), and placebo. Table S3 summarizes the characteristics of the included studies. All studies were industry sponsored and were judged to have an overall “low” risk of bias (Table S4). The examination of study characteristics across different trials demonstrated no evidence of transitivity violations (Table S2).

**Figure 1:**
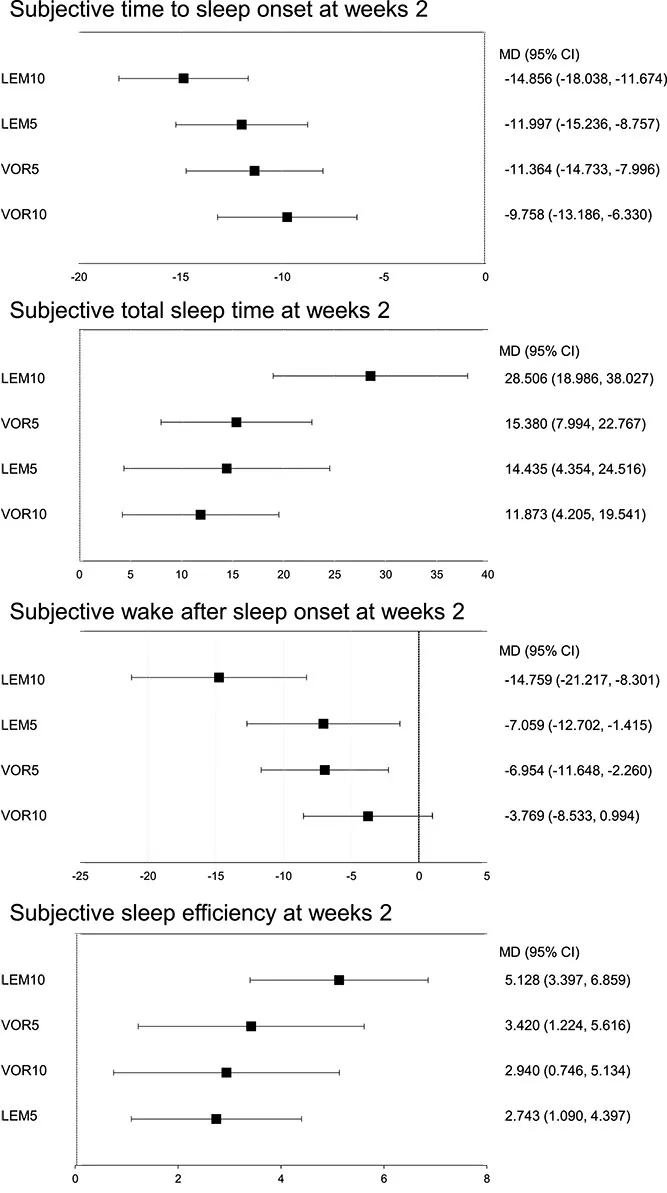
Forest plot. All efficacy outcomes are subjective endpoints. The primary outcome is sTSO at week 2. Abbreviations: 95% CI, 95% confidence interval; LEM5, lemborexant 5 mg/day; LEM10, lemborexant 10 mg/day; MD, mean difference; sSE, subjective sleep efficiency; sTSO, subjective time to sleep onset; sTST, subjective total sleep time; VOR5, vornorexant 5 mg/day; VOR10, vornorexant 10 mg/day; sWASO, subjective wake after sleep onset.

Supplementary Appendices S1–S12 detail the network meta-analysis results. All active treatments were significantly associated with sTSO improvements at week 2 compared with placebo (Figure 1 and Table 1). The mean difference (95% CI, min) in sTSO at week 2 ranged in LEM5, LEM10, VOR5, VOR10 were −11.997 (−15.236, −8.757), −14.856 (−18.038, −11.674), −11.364 (−14.733, −7.996), and − 9.758 (−13.186, −6.330), respectively. Network meta-regression analyses identified no potential confounding factors affecting sTSO effect sizes (Supplementary Appendix S1). LEM5, LEM10, and VOR5 demonstrated significant improvements across all other efficacy outcomes at weeks 1 and 2 over placebo (Figure 1 and Tables 1 and 2). Although VOR10 did not differ from placebo in sWASO at weeks 1 and 2, it was superior to placebo for all other efficacy outcomes (Figure 1 and Tables 1 and 2). LEM10 demonstrated the highest SUCRA values in terms of all efficacy outcomes (Supplementary Appendix S1–S8). However, these results should be interpreted as tentative, as they are significantly influenced by the limited nature of the indirect evidence within the network.

**Table 1:** League tables for efficacy outcomes at week 2.

A. sTSO at week 2 (minutes)				
**LEM10**	**−3.194 (−6.647, 0.259)**	**−4.755 (−9.702, 0.192)**	**−3.148 (−8.055, 1.758)**	**−14.856 (−18.038, −11.674)**
	LEM5	−1.679 (−6.573, 3.215)	−0.073 (−4.925, 4.780)	**−11.997 (−15.236, −8.757)**
		VOR10	2.395 (−2.937, 7.726)	**−9.758 (−13.186, −6.330)**
			VOR5	**−11.364 (−14.733, −7.996)**
				PLA
B. sTST at week 2 (minutes)				
**LEM10**	**15.275 (7.503, 23.047)**	**16.601 (5.614, 27.589)**	**13.094 (2.301, 23.887)**	**28.506 (18.986, 38.027)**
	LEM5	1.792 (−9.053, 12.637)	−1.716 (−12.364, 8.932)	**14.435 (4.354, 24.516)**
		VOR10	−3.191 (−10.475, 4.092)	**11.873 (4.205, 19.541)**
			VOR5	**15.380 (7.994, 22.767)**
				PLA
C. sWASO at week 2 (minutes)				
**LEM10**	**−8.281 (−14.705, −1.857)**	**−9.336 (−17.180, −1.492)**	**−6.151 (−13.952, 1.651)**	**−14.759 (−21.217, −8.301)**
	LEM5	−2.276 (−9.940, 5.387)	0.909 (−6.711, 8.529)	**−7.059 (−12.702, −1.415)**
		VOR10	3.483 (−2.184, 9151)	−3.769 (−8.533, 0.994)
			VOR5	**−6.954 (−11.648, −2.260)**
				PLA
D. sSE at week 2 (%)				
**LEM10**	**2.491 (0.987, 3.996)**	**2.119 (−0.611, 5.246)**	**1.791 (−1.023, 4.177)**	**5.128 (3.397, 6.859)**
	LEM5	−0.211 (−3.199, 2.550)	−0.701 (−4.011, 2.187)	**2.743 (1.090, 4.397)**
		VOR10	−0.480 (−2.018, 1.058)	**2.940 (0.746, 5.134)**
			VOR5	**3.420 (1.224, 5.616)**
				PLA

Abbreviations: LEM5, lemborexant 5 mg/day; LEM10, lemborexant 10 mg/day; sSE, subjective sleep efficiency; sTSO, subjective time to sleep onset; sTST, subjective total sleep time; sWASO, subjective wake after sleep onset; VOR5, vornorexant 5 mg/day; VOR10, vornorexant 10 mg/day

Each cell provides the mean difference (MD) with a 95% confidence interval for the corresponding comparison. Cells in bold indicate statistically significant results.

Comparisons are interpreted from left to right, with each estimate representing the difference between the column-defining and row-defining treatments. For sTSO and sWASO, MDs < 0 favor the row-defining treatment. Conversely, for sTST and sSE, MDs > 0 favor the row-defining treatment.

**Table 2:** League tables for efficacy outcomes at week 1.

A. sTSO at week 1 (minutes)				
**LEM10**	**−1.101 (−4.377, 2.175)**	**−6.057 (−10.788, −1.325)**	**−5.042 (−9.728, −0.357)**	**−15.656 (−18.613, −11.699)**
	LEM5	**−5.115 (−9.846, −0.384)**	−4.100 (−8.786, 0.585)	**−14.035 (−17.151, −10.918)**
		VOR10	0.955 (−2.325, 4.237)	**−8.847 (−12.232, −5.462)**
			VOR5	**−9.861 (−13.182, −6.541)**
				PLA
B. sTST at week 1 (minutes)				
**LEM10**	**13.860 (6.869, 20.950)**	**22.177 (12.265, 32.089)**	**19.359 (9.658, 29.060)**	**33.453 (26.949, 39.956)**
	LEM5	8.373 (−1.365, 18.110)	5.555 (−3.969, 15.078)	**19.483 (12.657, 26.309)**
		VOR10	−2.308 (−8.174, 4.559)	**11.043 (4.083, 18.003)**
			VOR5	**13.861 (7.204, 20.518)**
				PLA
C. sWASO at week 1 (minutes)				
**LEM10**	**−9.605 (−19.034, −0.177)**	**−15.204 (−23.918, −6.490)**	**−13.272 (−21.921, −4.624)**	**−20.605 (−26.363, −14.847)**
	LEM5	−7.633 (−16.133, 0.866)	−5.702 (−14.134, 2.731)	**−13.215 (−18.632, −7.799)**
		VOR10	1.615 (−4.291, 3.595)	−5.440 (−11.068, 0.188)
			VOR5	**−7.372 (−12.898, −1.846)**
				PLA
D. sSE at week 1 (%)				
**LEM10**	**2.348 (0.673, 4.023)**	**3.098 (1.068, 4.923)**	**3.012 (0.870, 5.101)**	**6.092 (4.827, 7.356)**
	LEM5	0.898 (−1.068, 2.850)	0.716 (−1.415, 2.545)	**3.892 (2.552, 5.223)**
		VOR10	−0.190 (−1.622, 1.242)	**3.000 (1.568, 4.432)**
			VOR5	**3.190 (1.755, 4.625)**
				PLA

Abbreviations: LEM5, lemborexant 5 mg/day; LEM10, lemborexant 10 mg/day; sSE, subjective sleep efficiency; sTSO, subjective time to sleep onset; sTST, subjective total sleep time; sWASO, subjective wake after sleep onset, VOR5, vornorexant 5 mg/day; VOR10, vornorexant 10 mg/day

Each cell provides the mean difference (MD) with a 95% confidence interval for the corresponding comparison. Cells in bold indicate statistically significant results.

Comparisons are interpreted from left to right, with each estimate representing the difference between the column-defining and row-defining treatments. For sTSO and sWASO, MDs < 0 favor the row-defining treatment. Conversely, for sTST and sSE, MDs > 0 favor the row-defining treatment.

Both LEM5 and LEM10 were associated with a higher incidence of at least one adverse event and somnolence compared with placebo (Supplementary Appendices S10 and S11). Conversely, neither LEM5 nor LEM10 was associated with an increased risk of adverse event-related discontinuation or headache (Supplementary Appendices S9 and S12). In contrast, VOR5 and VOR10 demonstrated no significant differences in tolerability or safety outcomes versus placebo. The incidence of death, suicidal behavior and ideation, fatigue, dizziness, falls, nightmares, cataplexy, and sleep paralysis was low, precluding their inclusion in the network meta-analysis (Supplementary Appendix S13).

Appendices S1–S12 present the results for heterogeneity and inconsistency for all outcomes. Global heterogeneity and inconsistency were not detected for any of the outcomes. Furthermore, analysis of pairwise comparisons revealed no evidence of considerable heterogeneity or statistically significant local inconsistency. Most comparisons were judged to have “some concerns” regarding within-study bias. Publication bias was not assessed because funnel plots are considered unreliable when fewer than 10 studies are available.^5^ Therefore, all comparisons for publication bias were rated as “some concerns.” Comparisons according to indirect evidence were downgraded by one level. Consequently, most evidence, including direct comparisons, was rated as having moderate certainty, whereas evidence derived solely from indirect comparisons was judged to have low or very low overall confidence.

## Discussion

This study is the first network meta-analysis to compare the efficacy, tolerability, and safety of VOR and LEM in adults with insomnia disorder, enabling indirect comparisons between these agents that have not been compared in clinical trials.

LEM10 exhibited the largest effect sizes across all efficacy outcomes at weeks 1 and 2. LEM5 and VOR5 demonstrated comparable efficacy across all outcomes at week 2; however, LEM5 exhibited numerically larger effect sizes than VOR5 for all efficacy outcomes at week 1. VOR is rapidly absorbed and has a t₁/₂ of approximately 2 h^1^; however, VOR5 improved sTST and sWASO. By facilitating sleep onset, VOR may reduce nocturnal awakenings, thereby contributing to an increase in total sleep time.

LEM exhibited a dose-dependent increase in effect sizes across efficacy outcomes when 10 and 5 mg doses were compared, and no corresponding dose–response pattern was observed for VOR. Notably, VOR 10 mg failed to demonstrate statistical superiority over placebo for sWASO. Thus, these findings suggest a potential dose-dependent effect for LEM, whereas no clear dose–response relationship was observed for VOR. However, these findings should be interpreted with caution owing to their exploratory nature, challenges in maintaining transitivity across trials with different treatment durations, limited number of available studies (particularly regarding VOR), and inherent uncertainty of indirect evidence.

Regarding safety, LEM, particularly LEM10, is associated with a higher incidence of somnolence compared with placebo. In contrast, no such signal was detected for VOR; however, this observation is based on only two studies, limiting the strength of the conclusions that can be drawn. Furthermore, analysis of several clinically important adverse events (eg, nightmares, cataplexy, sleep paralysis) was not feasible due to the scarcity or absence of events in the included trials; consequently, these outcomes were evaluated via single-group meta-analyses.

A phase 3 trial of VOR demonstrated no increased risk of withdrawal symptoms or rebound insomnia.^11^ Similarly, our previous systematic review^3^^,^^4^ demonstrated that LEM shares this favorable safety profile.

Our study has several limitations. First, our meta-analysis included a small number of studies and participants. Second, the observation period was limited to 2 weeks, consistent with the study duration of the VOR trial. Third, all comparisons between VOR and LEM were based on indirect evidence from a network meta-analysis. Fourth, this study did not address certain practical considerations relevant to clinical decision-making, including the integration of VOR or LEM with other pharmacological or nonpharmacological interventions and cost-effectiveness.

In conclusion, dosage variations may influence both the clinical efficacy and safety profile of these agents. Of note, VOR 10 mg failed to show a statistically significant advantage over placebo regarding sWASO. However, all comparisons between VOR and LEM were based on indirect evidence from a network meta-analysis; therefore, head-to-head trials comparing these agents in this population are warranted.

## Supplementary Material

Supplementary_material_final_pyag047

## Data Availability

The datasets generated during and/or analyzed during the current study are available from the corresponding author on reasonable request.
