# Supplementary material for: Vornorexant versus lemborexant for insomnia disorder: a systematic review and network meta-analysis
Source: Int J Neuropsychopharmacol. 2026 Sep 1;29(9):pyag047. doi: 10.1093/ijnp/pyag047 (PMC13580082; doi:10.1093/ijnp/pyag047)
Supplement: Supplementary file 1 [file supplementary_material_final_pyag047.docx]

**Figure S1. PRISMA 2020 flow diagram**

The “PICOS” strategy was used for the systematic literature review as follows: Participants were adults with insomnia disorder; Intervention was VOR and/or LEM; Control was a placebo; Outcomes included efficacy, tolerability, and safety; and Studies were double-blind, randomized, placebo-controlled trials. Inclusion criteria were as follows: (1) studies lasting at least 2 weeks that included VOR or LEM; (2) studies involving adult patients with insomnia, as defined by any recognized diagnostic criteria. Exclusion criteria were as follows: (1) studies involving pediatric or adolescent participants; (2) studies that included only an unrecommended dose arm in the United States,(FDA) Europe,(EMA) or Japan.(PMDA) A literature search of previous systematic reviews with inclusion criteria identical to those of the present study was conducted up to September 1, 2024.(Kishi et al., 2025) Subsequently, PubMed, Cochrane Library, and Embase databases were searched for studies published from January 1, 2024, to May 22, 2026, without language restriction. The search terms for PubMed and the Cochrane Library included (“vornorexant” OR “lemborexant”) AND (random*) AND (“insomnia”). The search terms for Embase included (“lemborexant”/exp OR “lemborexant” OR “vornorexant”/exp OR “vornorexant”) AND (“randomized controlled trial”/exp OR “randomized controlled trial”) AND (“insomnia”/exp OR “insomnia”). Reference lists of the selected research and review articles were manually searched for additional relevant published and unpublished studies, including conference abstracts. Furthermore, clinical trial registries (ClinicalTrials.gov, EU-CTR and WHO-ICTRP) were searched to ensure comprehensive trials identification and to minimize publication bias.Author consensus was utilized to resolve any discrepancies in the selected articles. Multiple papers or academic conference abstracts reporting the same research were screened by verifying the clinical trial registration number and/or reference to previous review articles.

1.	Kishi T, Ikuta T, Citrome L, Sakuma K, Hatano M, Hamanaka S et al. Comparative efficacy and safety of daridorexant, lemborexant, and suvorexant for insomnia: a systematic review and network meta-analysis. Transl Psychiatry 2025; 15(1): 211.

**Table S1. PRISMA for Network Meta-Analyses Checklist**

| **Section/Topic** | **Item #** | **Checklist Item** | **Reported on Page #** |
| --- | --- | --- | --- |
| **TITLE** |  |  |  |
| Title | 1 | Identify the report as a systematic review *incorporating a network meta-analysis (or related form of meta-analysis).* | 1 |
| **ABSTRACT** |  |  |  |
| Structured summary | 2 | Provide a structured summary including, as applicable:  **Background:** main objectives  **Methods:** data sources; study eligibility criteria, participants, and interventions; study appraisal; and *synthesis methods, such as network meta-analysis.*  **Results:** number of studies and participants identified; summary estimates with corresponding confidence/credible intervals; *treatment rankings may also be discussed. Authors may choose to summarize pairwise comparisons against a chosen treatment included in their analyses for brevity.*  **Discussion/Conclusions:** limitations; conclusions and implications of findings.  **Other:** primary source of funding; systematic review registration number with registry name. | 3- |
| **INTRODUCTION** |  |  |  |
| Rationale | 3 | Describe the rationale for the review in the context of what is already known*, including mention of why a network meta-analysis has been conducted.* | 5- |
| Objectives | 4 | Provide an explicit statement of questions being addressed, with reference to participants, interventions, comparisons, outcomes, and study design (PICOS). | 5- |
| **METHODS** |  |  |  |
| Protocol and registration | 5 | Indicate whether a review protocol exists and if and where it can be accessed (e.g., Web address); and, if available, provide registration information, including registration number. | 7- |
| Eligibility criteria | 6 | Specify study characteristics (e.g., PICOS, length of follow-up) and report characteristics (e.g., years considered, language, publication status) used as criteria for eligibility, giving rationale. *Clearly describe eligible treatments included in the treatment network, and note whether any have been clustered or merged into the same node (with justification).* | 7- |
| Information sources | 7 | Describe all information sources (e.g., databases with dates of coverage, contact with study authors to identify additional studies) in the search and date last searched. | 7- |
| Search | 8 | Present full electronic search strategy for at least one database, including any limits used, such that it could be repeated. | 7- |
| Study selection | 9 | State the process for selecting studies (i.e., screening, eligibility, included in systematic review, and, if applicable, included in the meta-analysis). | 7- |
| Data collection process | 10 | Describe method of data extraction from reports (e.g., piloted forms, independently, in duplicate) and any processes for obtaining and confirming data from investigators. | 7- |
| Data items | 11 | List and define all variables for which data were sought (e.g., PICOS, funding sources) and any assumptions and simplifications made. | 7- |
| **Geometry of the network** | **S1** | Describe methods used to explore the geometry of the treatment network under study and potential biases related to it. This should include how the evidence base has been graphically summarized for presentation, and what characteristics were compiled and used to describe the evidence base to readers. | 7- |
| Risk of bias within individual studies | 12 | Describe methods used for assessing risk of bias of individual studies (including specification of whether this was done at the study or outcome level), and how this information is to be used in any data synthesis. | 7- |
| Summary measures | 13 | State the principal summary measures (e.g., risk ratio, difference in means). *Also describe the use of additional summary measures assessed, such as treatment rankings and surface under the cumulative ranking curve (SUCRA)* values, as well as modified approaches used to present summary findings from meta-analyses.* | 7- |
| Planned methods of analysis | 14 | Describe the methods of handling data and combining results of studies for each network meta-analysis. This should include, but not be limited to:   - *Handling of multi-arm trials;* - *Selection of variance structure;* - *Selection of prior distributions in Bayesian analyses; and* - *Assessment of model fit.* | 7- |
| **Assessment of Inconsistency** | **S2** | Describe the statistical methods used to evaluate the agreement of direct and indirect evidence in the treatment network(s) studied. Describe efforts taken to address its presence when found. | 7- |
| Risk of bias across studies | 15 | Specify any assessment of risk of bias that may affect the cumulative evidence (e.g., publication bias, selective reporting within studies). | 7- |
| Additional analyses | 16 | Describe methods of additional analyses if done, indicating which were pre-specified. This may include, but not be limited to, the following:   - Sensitivity or subgroup analyses; - Meta-regression analyses; - *Alternative formulations of the treatment network; and* - *Use of alternative prior distributions for Bayesian analyses (if applicable).* | 7- |
| **RESULTS†** |  |  |  |
| Study selection | 17 | Give numbers of studies screened, assessed for eligibility, and included in the review, with reasons for exclusions at each stage, ideally with a flow diagram. | 10- |
| **Presentation of network structure** | **S3** | Provide a network graph of the included studies to enable visualization of the geometry of the treatment network. | 10- |
| **Summary of network geometry** | **S4** | Provide a brief overview of characteristics of the treatment network. This may include commentary on the abundance of trials and randomized patients for the different interventions and pairwise comparisons in the network, gaps of evidence in the treatment network, and potential biases reflected by the network structure. | 10- |
| Study characteristics | 18 | For each study, present characteristics for which data were extracted (e.g., study size, PICOS, follow-up period) and provide the citations. | 10- |
| Risk of bias within studies | 19 | Present data on risk of bias of each study and, if available, any outcome level assessment. | 10- |
| Results of individual studies | 20 | For all outcomes considered (benefits or harms), present, for each study: 1) simple summary data for each intervention group, and 2) effect estimates and confidence intervals. *Modified approaches may be needed to deal with information from larger networks.* | 10- |
| Synthesis of results | 21 | Present results of each meta-analysis done, including confidence/credible intervals. *In larger networks, authors may focus on comparisons versus a particular comparator (e.g. placebo or standard care), with full findings presented in an appendix. League tables and forest plots may be considered to summarize pairwise comparisons.* If additional summary measures were explored (such as treatment rankings), these should also be presented. | 10- |
| **Exploration for inconsistency** | **S5** | Describe results from investigations of inconsistency. This may include such information as measures of model fit to compare consistency and inconsistency models, *P* values from statistical tests, or summary of inconsistency estimates from different parts of the treatment network. | 10- |
| Risk of bias across studies | 22 | Present results of any assessment of risk of bias across studies for the evidence base being studied. | 10- |
| Results of additional analyses | 23 | Give results of additional analyses, if done (e.g., sensitivity or subgroup analyses, meta-regression analyses*, alternative network geometries studied, alternative choice of prior distributions for Bayesian analyses,* and so forth). | 10- |
| **DISCUSSION** |  |  |  |
| Summary of evidence | 24 | Summarize the main findings, including the strength of evidence for each main outcome; consider their relevance to key groups (e.g., healthcare providers, users, and policy-makers). | 14- |
| Limitations | 25 | Discuss limitations at study and outcome level (e.g., risk of bias), and at review level (e.g., incomplete retrieval of identified research, reporting bias). *Comment on the validity of the assumptions, such as transitivity and consistency. Comment on any concerns regarding network geometry (e.g., avoidance of certain comparisons).* | 14- |
| Conclusions | 26 | Provide a general interpretation of the results in the context of other evidence, and implications for future research. | 14- |
|  |  |  |  |
| **FUNDING** |  |  | 19 |
| Funding | 27 | Describe sources of funding for the systematic review and other support (e.g., supply of data); role of funders for the systematic review. This should also include information regarding whether funding has been received from manufacturers of treatments in the network and/or whether some of the authors are content experts with professional conflicts of interest that could affect use of treatments in the network. |  |

**Table S2. Transitivity assessment**

|  | Boxplot | Kruskal–Wallis equality of populations rank test. |
| --- | --- | --- |
| Proportion of females (k = 7) |  | Chi-squared with ties = 6.07 (df = 4), p = 0.110 |
| Mean age (k = 7) |  | Chi-squared with ties = 0.45 (df = 4), p = 0.979 |
| Proportion of elderly people (aged ≥65 years) (k = 6) |  | Chi-squared with ties = 0.38 (df = 4), p = 0.984 |
| Total number of participants (k = 7) |  | Chi-squared with ties = 0.96 (df = 7), p = 0.916 |

**Table S3. Characteristics of studies included in the systematic review and network meta-analysis**

| **Study**  [1] study name (trial ID), [2] treatment (n), [3] study duration | **Inclusion criteria**  [1] diagnosis, [2] key inclusion criteria, [3] PLA lead-in period | **Characteristics of participants**  [1] mean age (SD), %people≥65 years, [2] %female, [3] race/ethnicity |
| --- | --- | --- |
| [1] Murphy 2017 (NCT01995838)  [2] LEM5 (38), LEM10 (32), PLA (56)  [3] 2 weeks | [1] insomnia disorder (DSM-5)  [2] male and female 19 to 80 years, TSO mean ≥ 30 minutes, WASO mean ≥ 30 minute and SE mean ≤ 85% on 2 consecutive PSGs  [3] yes | [1] 48.3 (14.8) years, 17.5%  [2] 62.7%  [3] Caucasian: 73.0% |
| [1] Rosenberg 2019 (NCT02783729)  [2] LEM5 (266), LEM10 (269), PLA (208)  [3] 1 month | [1] insomnia disorder (DSM-5)  [2] male ≥ 65 years and female ≥ 55 years, sWASO ≥ 60 minutes for ≥ 3 nights/week, ISI ≥ 13, and WASO mean ≥ 60 minutes on 2 consecutive PSGs with neither night < 45 minutes.  [3] yes | [1] 63.8 (6.7) years, 44.8%  [2] 86.5%  [3] Caucasian: 74.6% |
| [1] Kärppä 2020 (NCT02952820)  [2] LEM 5 (323), LEM 10 (323), PLA (325)  [3] 6 months* | [1] insomnia disorder (DSM-5)  [2] male and female ≥ 18 years, sTSO ≥ 30 minutes and/or sWASO ≥ 60 minutes for ≥ 3 nights/week, and ISI ≥ 15.  [3] yes | [1] 54.5 (13.8) years, 27.6%  [2] 68.2%  [3] Caucasian: 71.5% |
| [1] Mi 2025 (NCT04549168)  [2] LEM10 (94), PLA (100)  [3] 1 month | [1] insomnia disorder (DSM-5)  [2] male and female ≥ 18 years, sTSO ≥ 30 minutes and/or sWASO ≥ 60 minutes for ≥ 3 nights/week, and ISI ≥ 15.  [3] yes | [1] 42.5 (13.1) years, 5.2%  [2] 68.0%  [3] Chinese: 100.0% |
| [1] NCT05594589  [2] LEM 5 (26), LEM 10 (26), PLA (13)  [3] 1 month | [1] insomnia disorder (DSM-5)  [2] male and female 19 to 80 years sTSO ≥ 30 minutes for ≥ 3 nights/week, and ISI ≥ 13.  [3] yes | [1] 46.2 (15.0) years, 12.3%  [2] 61.5%  [3] Korean: 100.0% |
| [1] Uchiyama 2025 (NCT05453136)  [2] VOR5 (196), VOR10 (197), PLA (197)  [3] 2 weeks | [1] insomnia disorder (DSM-5)  [2] male and female ≥ 18 years, sTSO ≥ 30 minutes and sTST < 6.5 hours for ≥ 4 nights/week, and ISI ≥ 15.  [3] yes | [1] 53.4 (12.9) years, 21.9%  [2] 54.3%  [3] Japanese: 100.0% |
| [1] TS142-203 (NCT04469023)  [2] VOR5 (45), VOR10 (44), PLA (45)  [3] 2 weeks | [1] insomnia disorder (DSM-5)  [2] male and female ≥ 20 years, sTSO ≥ 30 minutes and sTST < 6.5 hours for ≥ 4 nights/week, and ISI ≥ 15.  [3] yes | [1] 47.7 (NR) years, ** NR  [2] 57.3%**  [3] Japanese: 100.0% |

*Based on the 6-month double-blind, randomized, placebo-controlled trial (period 1), patients in the placebo group were re-randomized to LEM 5 mg or LEM 10 mg until month 12 (period 2). Data from period 1 were used in the present study.

**This study included VOR2.5, VOR5, VOR10, and placebo arms. Participant characteristics were derived from the total sample across all treatment arms, as these data were not reported separately for each individual arm.

DSM: Diagnostic and Statistical Manual of Mental Disorders, ISI: Insomnia Severity Index, LEM: lemborexant, n: number of participants, NR: not report, PLA: placebo, PSG: polysomnography, SD: standard deviation, sTSO: subjective time to sleep onset, sTST: subjective total sleep time, sWASO: subjective wake-after-sleep onset, VOR5: vornorexant 5 mg/day, VOR10: vornorexant 10 mg/day

**Table S4. Risk of bias summary**

|  | Randomization process | Deviation from intended intervention | Missing outcome data | Measurement of the outcome | Selection of the reported result | Overall risk of bias |
| --- | --- | --- | --- | --- | --- | --- |
| Murphy 2017 (NCT01995838) | Low risk | Low risk | Low risk | Low risk | Low risk | Low risk |
| Rosenberg 2019 (NCT02783729) | Low risk | Low risk | Low risk | Low risk | Low risk | Low risk |
| Kärppä 2020 (NCT02952820) | Low risk | Low risk | Low risk | Low risk | Low risk | Low risk |
| Mi 2025 (NCT04549168) | Low risk | Low risk | Low risk | Low risk | Low risk | Low risk |
| NCT05594589 | Low risk | Low risk | Low risk | Low risk | Low risk | Low risk |
| Uchiyama 2025 (NCT05453136) | Low risk | Low risk | Low risk | Low risk | Low risk | Low risk |
| TS142-203 (NCT04469023) | Low risk | Low risk | Low risk | Low risk | Low risk | Low risk |

**Appendix S1. Subjective time to sleep onset at week 2**

6 studies, 2672 participants

A frequentist network meta-analysis with a random-effects model was conducted to mean differences and odds ratios with corresponding 95% confidence intervals [1, 2].

[1] DerSimonian R, Laird N. Meta-analysis in clinical trials. Controlled clinical trials. 1986;7:177-88.

[2] Rücker G, Schwarzer G, Krahn U, König J. netmeta: Network Meta-Analysis using Frequentist Methods (R package version 0.9-5). https://CRANR-projectorg/package=netmeta. 2017;(accessed March 14, 2020).

**League table (MD with 95% CI)**

| LEM10 | -3.194 (-6.647, 0.259) | -4.755 (-9.702, 0.192) | -3.148 (-8.055, 1.758) | **-14.856 (-18.038, -11.674)** |
| --- | --- | --- | --- | --- |
|  | LEM5 | -1.679 (-6.573, 3.215) | -0.073 (-4.925, 4.780) | **-11.997 (-15.236, -8.757)** |
|  |  | VOR10 | 2.395 (-2.937, 7.726) | **-9.758 (-13.186, -6.330)** |
|  |  |  | VOR5 | **-11.364 (-14.733, -7.996)** |
|  |  |  |  | PLA |

**SUCRA**

| LEM10 | 98.5% |
| --- | --- |
| LEM5 | 85.9% |
| VOR10 | 75.2% |
| VOR5 | 84.0% |
| PLA | 0.8% |

**Global heterogeneity**

As previously suggested (Huhn 2019), the common 𝜏^2^ was compared to the empirical distributions of heterogeneity found in the meta‐analyses of pharmacological treatments for mental health outcomes, with a median of the 𝜏^2^ distribution of 0.049 and an inter‐quartile range of 0.010 to 0.242 (Rhodes 2015), and the heterogeneity was considered low when the estimated 𝜏^2^ was below the 25% quartile, moderate when between 25% and 50% of the quartile, and high when above the 50% quartile.

Huhn M, et al. Lancet 2019;394(10202):939-51

Rhodes KM, et al. J Clin Epidemiol 2015;68(1):52-60

Between study variance (𝜏^2^): 0.023 (heterogeneity assessment: low)

**Random-effects design-by-treatment interaction model**

P value: 0.244

**Local heterogeneity (I^2^) and incoherence (SIDE test)**

I^2^ ≥ 50% was considered as considerable heterogeneity.

As a general rule, there are “no concerns” if the p-value is >0.10, independent of the position of the 95% CIs with respect to the range of equivalence, because the evidence for incoherence is weak (p > 0.10).

Nikolakopoulou A, et al., PLOS Medicine 2020 17 1-19, Papakonstantinou T, et al., Campbell Systematic Reviews 2020 16 e1080

|  | I^2^ (%) | P value (SIDE test) |
| --- | --- | --- |
| LEM10 vs LEM5 | 0.0 | No inconsistency |
| LEM10 vs PLA | 0.0 | No inconsistency |
| LEM5 vs PLA | 0.0 | No inconsistency |
| VOR10 vs VOR5 | 45.2 | No inconsistency |
| VOR10 vs PLA | 0.0 | No inconsistency |
| VOR5 vs PLA | 0.0 | No inconsistency |

**Funnel plot (all drugs vs. placebo)**

As none of the comparisons included 10 or more studies, the assessment of publication bias was not performed.

**Meta-regression analysis (the placebo was the control)**

| Potential moderators | β (SE) |
| --- | --- |
| Mean age (k = 6) | -0.101 (3.023) |
| Proportion of females (k = 6) | -0.012 (0.252) |
| Proportion of elderly patients (k = 5) | -2.434 (5.999) |
| Total participants (k = 6) | 2.007 (5.933) |

van Valkenhoef G, Lu G, de Brock B, Hillege H, Ades AE, Welton NJ. Automating network meta-analysis. Res Synth Methods. 2012;3:285-99.

**CINeMA confidence rating**

CINeMA is a web application that simplifies the evaluation of confidence in the findings from a network meta-analysis. CINeMA is based on a methodological framework described in the following articles, which consider the following six domains: within-study bias, reporting bias, indirectness, imprecision, heterogeneity, and incoherence. CINeMA grades the confidence in the results of each treatment comparison as high, moderate, low, or very low. If the comparison had only indirect evidence, the comparison was downgraded one level.

Nikolakopoulou A, et al., PLOS Medicine 2020 17 1-19, Papakonstantinou T, et al., Campbell Systematic Reviews 2020 16 e1080

(1) Within-study bias: Risk of bias in RCTs for the main outcomes was assessed independently using the Cochrane risk-of-bias tool for randomized trials (RoB 2). In all studies, the overall risk of bias was evaluated as “low risk.” Selected rule: Average

(2) Reporting bias: Comparison-adjusted funnel plots with less than 10 studies are not meaningful. Therefore, all comparisons were “Suspected.”

(3) Indirectness: No indirectness was assumed. Selected rule: Average

(4) Imprecision: For placebo comparisons the clinically meaningful threshold was set at a OR of higher or lower than 1. For placebo comparisons the clinically meaningful threshold was set at a SMD of higher or lower than 0. For comparisons of two active treatments the clinically meaningful threshold was set at SMDs of -0.1 and 0.1, and at ORs of 0.8 and 1.25

for dichotomous outcomes.

(5) Heterogeneity: We used recommendations automatically provided by CINeMA.

(6) Incoherence: We used recommendations automatically provided by CINeMA.

| Comparison | Number of studies | Within-study bias | Reporting bias | Indirectness | Imprecision | Heterogeneity | Incoherence | Confidence rating |
| --- | --- | --- | --- | --- | --- | --- | --- | --- |
| LEM10 vs LEM5 | 3 | No concerns | Some concerns | No concerns | No concerns | No concerns | No concerns | Moderate |
| LEM10 vs PLA | 4 | No concerns | Some concerns | No concerns | No concerns | No concerns | No concerns | Moderate |
| LEM5 vs PLA | 4 | No concerns | Some concerns | No concerns | No concerns | No concerns | No concerns | Moderate |
| VOR10 vs VOR5 | 2 | No concerns | Some concerns | No concerns | No concerns | No concerns | No concerns | Moderate |
| VOR10 vs PLA | 2 | No concerns | Some concerns | No concerns | No concerns | No concerns | No concerns | Moderate |
| VOR5 vs PLA | 2 | No concerns | Some concerns | No concerns | No concerns | No concerns | No concerns | Moderate |
| LEM10 vs VOR10 | 0 | No concerns | Some concerns | No concerns | Major concerns | No concerns | Major concerns | Very low |
| LEM10 vs VOR5 | 0 | No concerns | Some concerns | No concerns | Major concerns | No concerns | Major concerns | Very low |
| LEM5 vs VOR10 | 0 | No concerns | Some concerns | No concerns | Major concerns | No concerns | Major concerns | Very low |
| LEM5 vs VOR5 | 0 | No concerns | Some concerns | No concerns | Major concerns | No concerns | Major concerns | Very low |

**Appendix S2.** **Subjective total sleep time at week 2**

6 studies, 2468 participants

**League table (MD with 95% CI)**

| LEM10 | **15.275 (7.503, 23.047)** | **16.601 (5.614, 27.589)** | **13.094 (2.301, 23.887)** | **28.506 (18.986, 38.027)** |
| --- | --- | --- | --- | --- |
|  | LEM5 | 1.792 (-9.053, 12.637) | -1.716 (-12.364, 8.932) | **14.435 (4.354, 24.516)** |
|  |  | VOR10 | -3.191 (-10.475, 4.092) | **11.873 (4.205, 19.541)** |
|  |  |  | VOR5 | **15.380 (7.994, 22.767)** |
|  |  |  |  | PLA |

**SUCRA**

| LEM10 | 98.6% |
| --- | --- |
| LEM5 | 80.9% |
| VOR10 | 74.2% |
| VOR5 | 81.2% |
| PLA | 0.9% |

**Global heterogeneity**

Between study variance (𝜏^2^): 0.000 (heterogeneity assessment: low)

**Random-effects design-by-treatment interaction model**

P value: 0.757

**Local heterogeneity (I^2^) and incoherence (SIDE test)**

|  | I^2^ (%) | P value (SIDE test) |
| --- | --- | --- |
| LEM10 vs LEM5 | 0.0 | No inconsistency |
| LEM10 vs PLA | 35.6 | No inconsistency |
| LEM5 vs PLA | 43.1 | No inconsistency |
| VOR10 vs VOR5 | 0.0 | No inconsistency |
| VOR10 vs PLA | 0.0 | No inconsistency |
| VOR5 vs PLA | 0.0 | No inconsistency |

**CINeMA confidence rating**

| Comparison | Number of studies | Within-study bias | Reporting bias | Indirectness | Imprecision | Heterogeneity | Incoherence | Confidence rating |
| --- | --- | --- | --- | --- | --- | --- | --- | --- |
| LEM10 vs LEM5 | 2 | No concerns | Some concerns | No concerns | No concerns | No concerns | No concerns | Moderate |
| LEM10 vs PLA | 3 | No concerns | Some concerns | No concerns | No concerns | No concerns | No concerns | Moderate |
| LEM5 vs PLA | 3 | No concerns | Some concerns | No concerns | No concerns | No concerns | No concerns | Moderate |
| VOR10 vs VOR5 | 2 | No concerns | Some concerns | No concerns | No concerns | No concerns | No concerns | Moderate |
| VOR10 vs PLA | 2 | No concerns | Some concerns | No concerns | No concerns | No concerns | No concerns | Moderate |
| VOR5 vs PLA | 2 | No concerns | Some concerns | No concerns | No concerns | No concerns | No concerns | Moderate |
| LEM10 vs VOR10 | 0 | No concerns | Some concerns | No concerns | No concerns | Major concerns | Major concerns | Very low |
| LEM10 vs VOR5 | 0 | No concerns | Some concerns | No concerns | No concerns | Major concerns | Major concerns | Very low |
| LEM5 vs VOR10 | 0 | No concerns | Some concerns | No concerns | Major concerns | No concerns | Major concerns | Very low |
| LEM5 vs VOR5 | 0 | No concerns | Some concerns | No concerns | Major concerns | No concerns | Major concerns | Very low |

**Appendix S3.** **Subjective wake after sleep onset at week 2**

6 studies, 2655 participants

**League table (MD with 95% CI)**

| LEM10 | **-8.281 (-14.705, -1.857)** | **-9.336 (-17.180, -1.492)** | -6.151 (-13.952, 1.651) | **-14.759 (-21.217, -8.301)** |
| --- | --- | --- | --- | --- |
|  | LEM5 | -2.276 (-9.940, 5.387) | 0.909 (-6.711, 8.529) | **-7.059 (-12.702, -1.415)** |
|  |  | VOR10 | 3.483 (-2.184, 9,151) | -3.769 (-8.533, 0.994) |
|  |  |  | VOR5 | **-6.954 (-11.648, -2.260)** |
|  |  |  |  | PLA |

**SUCRA**

| LEM10 | 98.7% |
| --- | --- |
| LEM5 | 81.7% |
| VOR10 | 52.8% |
| VOR5 | 79.9% |
| PLA | 1.0% |

**Global heterogeneity**

Between study variance (𝜏^2^): 0.000 (heterogeneity assessment: low)

**Random-effects design-by-treatment interaction model**

P value: 0.621

**Local heterogeneity (I^2^) and incoherence (SIDE test)**

|  | I^2^ (%) | P value (SIDE test) |
| --- | --- | --- |
| LEM10 vs LEM5 | 3.7 | No inconsistency |
| LEM10 vs PLA | 21.6 | No inconsistency |
| LEM5 vs PLA | 0.0 | No inconsistency |
| VOR10 vs VOR5 | 11.1 | No inconsistency |
| VOR10 vs PLA | 0.0 | No inconsistency |
| VOR5 vs PLA | 0.0 | No inconsistency |

**CINeMA confidence rating**

| Comparison | Number of studies | Within-study bias | Reporting bias | Indirectness | Imprecision | Heterogeneity | Incoherence | Confidence rating |
| --- | --- | --- | --- | --- | --- | --- | --- | --- |
| LEM10 vs LEM5 | 3 | No concerns | Some concerns | No concerns | No concerns | No concerns | No concerns | Moderate |
| LEM10 vs PLA | 4 | No concerns | Some concerns | No concerns | No concerns | No concerns | No concerns | Moderate |
| LEM5 vs PLA | 4 | No concerns | Some concerns | No concerns | No concerns | No concerns | No concerns | Moderate |
| VOR10 vs VOR5 | 2 | No concerns | Some concerns | No concerns | Major concerns | No concerns | No concerns | Low |
| VOR10 vs PLA | 2 | No concerns | Some concerns | No concerns | Major concerns | No concerns | No concerns | Low |
| VOR5 vs PLA | 2 | No concerns | Some concerns | No concerns | No concerns | No concerns | No concerns | Moderate |
| LEM10 vs VOR10 | 0 | No concerns | Some concerns | No concerns | No concerns | Major concerns | Major concerns | Very low |
| LEM10 vs VOR5 | 0 | No concerns | Some concerns | No concerns | Major concerns | No concerns | Major concerns | Very low |
| LEM5 vs VOR10 | 0 | No concerns | Some concerns | No concerns | Major concerns | No concerns | Major concerns | Very low |
| LEM5 vs VOR5 | 0 | No concerns | Some concerns | No concerns | Major concerns | No concerns | Major concerns | Very low |

**Appendix S4.** **Subjective sleep efficiency at week 2**

4 studies, 2334 participants

**League table (MD with 95% CI)**

| LEM10 | **2.491 (0.987, 3.996)** | 2.119 (-0.611, 5.246) | 1.791 (-1.023, 4.177) | **5.128 (3.397, 6.859)** |
| --- | --- | --- | --- | --- |
|  | LEM5 | -0.211 (-3.199, 2.550) | -0.701 (-4.011, 2.187) | **2.743 (1.090, 4.397)** |
|  |  | VOR10 | -0.480 (-2.018, 1.058) | **2.940 (0.746, 5.134)** |
|  |  |  | VOR5 | **3.420 (1.224, 5.616)** |
|  |  |  |  | PLA |

**SUCRA**

| LEM10 | 96.2% |
| --- | --- |
| LEM5 | 70.9% |
| VOR10 | 75.8% |
| VOR5 | 85.1% |
| PLA | 9.9% |

**Global heterogeneity**

Between study variance (𝜏^2^): 0.000 (heterogeneity assessment: low to moderate)

**Random-effects design-by-treatment interaction model**

P value: 0.746

**Local heterogeneity (I^2^) and incoherence (SIDE test)**

|  | I^2^ (%) | P value (SIDE test) |
| --- | --- | --- |
| LEM10 vs LEM5 | 0.0 | No inconsistency |
| LEM10 vs PLA | 28.5 | No inconsistency |
| LEM5 vs PLA | 21.3 | No inconsistency |

**CINeMA confidence rating**

| Comparison | Number of studies | Within-study bias | Reporting bias | Indirectness | Imprecision | Heterogeneity | Incoherence | Confidence rating |
| --- | --- | --- | --- | --- | --- | --- | --- | --- |
| LEM10 vs LEM5 | 2 | No concerns | Some concerns | No concerns | No concerns | No concerns | No concerns | Moderate |
| LEM10 vs PLA | 3 | No concerns | Some concerns | No concerns | No concerns | No concerns | No concerns | Moderate |
| LEM5 vs PLA | 3 | No concerns | Some concerns | No concerns | No concerns | No concerns | No concerns | Moderate |
| VOR10 vs VOR5 | 1 | No concerns | Some concerns | No concerns | Major concerns | No concerns | Major concerns | Low |
| VOR10 vs PLA | 1 | No concerns | Some concerns | No concerns | No concerns | Major concerns | Major concerns | Low |
| VOR5 vs PLA | 1 | No concerns | Some concerns | No concerns | No concerns | Major concerns | Major concerns | Low |
| LEM10 vs VOR10 | 0 | No concerns | Some concerns | No concerns | Major concerns | No concerns | Major concerns | Very low |
| LEM10 vs VOR5 | 0 | No concerns | Some concerns | No concerns | Major concerns | No concerns | Major concerns | Very low |
| LEM5 vs VOR10 | 0 | No concerns | Some concerns | No concerns | Major concerns | No concerns | Major concerns | Very low |
| LEM5 vs VOR5 | 0 | No concerns | Some concerns | No concerns | Major concerns | No concerns | Major concerns | Very low |

**Appendix S5. Subjective time to sleep onset at week 1**

6 studies, 2701 participants

**League table (MD with 95% CI)**

| LEM10 | -1.101 (-4.377, 2.175) | **-6.057 (-10.788, -1.325)** | -**5.042 (-9.728, -0.357)** | **-15.656 (-18.613, -11.699)** |
| --- | --- | --- | --- | --- |
|  | LEM5 | **-5.115 (-9.846, -0.384)** | -4.100 (-8.786, 0.585) | **-14.035 (-17.151, -10.918)** |
|  |  | VOR10 | 0.955 (-2.325, 4.237) | **-8.847 (-12.232, -5.462)** |
|  |  |  | VOR5 | **-9.861 (-13.182, -6.541)** |
|  |  |  |  | PLA |

**SUCRA**

| LEM10 | 96.1% |
| --- | --- |
| LEM5 | 93.9% |
| VOR10 | 72.3% |
| VOR5 | 75.9% |
| PLA | 1.2% |

**Global heterogeneity**

Between study variance (𝜏^2^): 0.000 (heterogeneity assessment: low)

**Random-effects design-by-treatment interaction model**

P value: 0.489

**Local heterogeneity (I^2^) and incoherence (SIDE test)**

|  | I^2^ (%) | P value (SIDE test) |
| --- | --- | --- |
| LEM10 vs LEM5 | 0.0 | No inconsistency |
| LEM10 vs PLA | 0.0 | No inconsistency |
| LEM5 vs PLA | 0.0 | No inconsistency |
| VOR10 vs VOR5 | 0.0 | No inconsistency |
| VOR10 vs PLA | 0.0 | No inconsistency |
| VOR5 vs PLA | 0.0 | No inconsistency |

**CINeMA confidence rating**

| Comparison | Number of studies | Within-study bias | Reporting bias | Indirectness | Imprecision | Heterogeneity | Incoherence | Confidence rating |
| --- | --- | --- | --- | --- | --- | --- | --- | --- |
| LEM10 vs LEM5 | 3 | No concerns | Some concerns | No concerns | Major concerns | No concerns | No concerns | Low |
| LEM10 vs PLA | 4 | No concerns | Some concerns | No concerns | No concerns | No concerns | No concerns | Moderate |
| LEM5 vs PLA | 4 | No concerns | Some concerns | No concerns | No concerns | No concerns | No concerns | Moderate |
| VOR10 vs VOR5 | 2 | No concerns | Some concerns | No concerns | Major concerns | No concerns | No concerns | Low |
| VOR10 vs PLA | 2 | No concerns | Some concerns | No concerns | No concerns | No concerns | No concerns | Moderate |
| VOR5 vs PLA | 2 | No concerns | Some concerns | No concerns | No concerns | No concerns | No concerns | Moderate |
| LEM10 vs VOR10 | 0 | No concerns | Some concerns | No concerns | No concerns | Major concerns | Major concerns | Very low |
| LEM10 vs VOR5 | 0 | No concerns | Some concerns | No concerns | No concerns | Major concerns | Major concerns | Very low |
| LEM5 vs VOR10 | 0 | No concerns | Some concerns | No concerns | No concerns | Major concerns | Major concerns | Very low |
| LEM5 vs VOR5 | 0 | No concerns | Some concerns | No concerns | Major concerns | No concerns | Major concerns | Very low |

**Appendix S6. Subjective total sleep time at week 1**

5 studies, 2503 participants

**League table (MD with 95% CI)**

| LEM10 | **13.860 (6.869, 20.950)** | **22.177 (12.265, 32.089)** | **19.359 (9.658, 29.060)** | **33.453 (26.949, 39.956)** |
| --- | --- | --- | --- | --- |
|  | LEM5 | 8.373 ( -1.365, 18.110) | 5.555 (-3.969, 15.078) | **19.483 (12.657, 26.309)** |
|  |  | VOR10 | -2.308 (-8.174, 4.559) | **11.043 (4.083, 18.003)** |
|  |  |  | VOR5 | **13.861 (7.204, 20.518)** |
|  |  |  |  | PLA |

**SUCRA**

| LEM10 | 96.9% |
| --- | --- |
| LEM5 | 85.3% |
| VOR10 | 72.9% |
| VOR5 | 77.1% |
| PLA | 0.6% |

**Global heterogeneity**

Between study variance (𝜏^2^): 0.000 (heterogeneity assessment: low)

**Random-effects design-by-treatment interaction model**

P value: 0.881

**Local heterogeneity (I^2^) and incoherence (SIDE test)**

|  | I^2^ (%) | P value (SIDE test) |
| --- | --- | --- |
| LEM10 vs LEM5 | 0.0 | No inconsistency |
| LEM10 vs PLA | 0.0 | No inconsistency |
| LEM5 vs PLA | 0.0 | No inconsistency |
| VOR10 vs VOR5 | 0.0 | No inconsistency |
| VOR10 vs PLA | 28.7 | No inconsistency |
| VOR5 vs PLA | 0.0 | No inconsistency |

**CINeMA confidence rating**

| Comparison | Number of studies | Within-study bias | Reporting bias | Indirectness | Imprecision | Heterogeneity | Incoherence | Confidence rating |
| --- | --- | --- | --- | --- | --- | --- | --- | --- |
| LEM10 vs LEM5 | 2 | No concerns | Some concerns | No concerns | No concerns | No concerns | No concerns | Moderate |
| LEM10 vs PLA | 3 | No concerns | Some concerns | No concerns | No concerns | No concerns | No concerns | Moderate |
| LEM5 vs PLA | 3 | No concerns | Some concerns | No concerns | No concerns | No concerns | No concerns | Moderate |
| VOR10 vs VOR5 | 2 | No concerns | Some concerns | No concerns | Major concerns | No concerns | No concerns | Low |
| VOR10 vs PLA | 2 | No concerns | Some concerns | No concerns | No concerns | No concerns | No concerns | Moderate |
| VOR5 vs PLA | 2 | No concerns | Some concerns | No concerns | No concerns | No concerns | No concerns | Moderate |
| LEM10 vs VOR10 | 0 | No concerns | Some concerns | No concerns | No concerns | No concerns | Major concerns | Very low |
| LEM10 vs VOR5 | 0 | No concerns | Some concerns | No concerns | No concerns | No concerns | Major concerns | Very low |
| LEM5 vs VOR10 | 0 | No concerns | Some concerns | No concerns | Major concerns | No concerns | Major concerns | Very low |
| LEM5 vs VOR5 | 0 | No concerns | Some concerns | No concerns | Major concerns | No concerns | Major concerns | Very low |

**Appendix S7. Subjective wake after sleep onset at week 1**

6 studies, 2690 participants

**League table (MD with 95% CI)**

| LEM10 | **-9.605 (-19.034, -0.177)** | **-15.204 (-23.918, -6.490)** | **-13.272 (-21.921, -4.624)** | **-20.605 (-26.363, -14.847)** |
| --- | --- | --- | --- | --- |
|  | LEM5 | -7.633 (-16.133, 0.866) | -5.702 (-14.134, 2.731) | **-13.215 (-18.632, -7.799)** |
|  |  | VOR10 | 1.615 (-4.291, 3.595) | -5.440 (-11.068, 0.188) |
|  |  |  | VOR5 | **-7.372 (-12.898, -1.846)** |
|  |  |  |  | PLA |

**SUCRA**

| LEM10 | 99.3% |
| --- | --- |
| LEM5 | 91.1% |
| VOR10 | 48.5% |
| VOR5 | 73.9% |
| PLA | 3.4% |

**Global heterogeneity**

Between study variance (𝜏^2^): 0.000 (heterogeneity assessment: low)

**Random-effects design-by-treatment interaction model**

P value: 0.590

**Local heterogeneity (I^2^) and incoherence (SIDE test)**

|  | I^2^ (%) | P value (SIDE test) |
| --- | --- | --- |
| LEM10 vs LEM5 | 49.2 | No inconsistency |
| LEM10 vs PLA | 14.6 | No inconsistency |
| LEM5 vs PLA | 0.0 | No inconsistency |
| VOR10 vs VOR5 | 0.0 | No inconsistency |
| VOR10 vs PLA | 0.0 | No inconsistency |
| VOR5 vs PLA | 0.0 | No inconsistency |

**CINeMA confidence rating**

| Comparison | Number of studies | Within-study bias | Reporting bias | Indirectness | Imprecision | Heterogeneity | Incoherence | Confidence rating |
| --- | --- | --- | --- | --- | --- | --- | --- | --- |
| LEM10 vs LEM5 | 3 | No concerns | Some concerns | No concerns | No concerns | No concerns | No concerns | Moderate |
| LEM10 vs PLA | 4 | No concerns | Some concerns | No concerns | No concerns | No concerns | No concerns | Moderate |
| LEM5 vs PLA | 4 | No concerns | Some concerns | No concerns | No concerns | No concerns | No concerns | Moderate |
| VOR10 vs VOR5 | 2 | No concerns | Some concerns | No concerns | Major concerns | No concerns | No concerns | Low |
| VOR10 vs PLA | 2 | No concerns | Some concerns | No concerns | Major concerns | No concerns | No concerns | Low |
| VOR5 vs PLA | 2 | No concerns | Some concerns | No concerns | No concerns | No concerns | No concerns | Moderate |
| LEM10 vs VOR10 | 0 | No concerns | Some concerns | No concerns | No concerns | Major concerns | Major concerns | Very low |
| LEM10 vs VOR5 | 0 | No concerns | Some concerns | No concerns | No concerns | Major concerns | Major concerns | Very low |
| LEM5 vs VOR10 | 0 | No concerns | Some concerns | No concerns | Major concerns | No concerns | Major concerns | Very low |
| LEM5 vs VOR5 | 0 | No concerns | Some concerns | No concerns | Major concerns | No concerns | Major concerns | Very low |

**Appendix S8. Subjective sleep efficiency at week 1**

4 studies, 2369 participants

**League table (MD with 95% CI)**

| LEM10 | **2.348 (0.673, 4.023)** | **3.098 (1.068, 4.923)** | **3.012 (0.870, 5.101)** | **6.092 (4.827, 7.356)** |
| --- | --- | --- | --- | --- |
|  | LEM5 | 0.898 (-1.068, 2.850) | 0.716 (-1.415, 2.545) | **3.892 (2.552, 5.223)** |
|  |  | VOR10 | -0.190 (-1.622, 1.242) | **3.000 (1.568, 4.432)** |
|  |  |  | VOR5 | **3.190 (1.755, 4.625)** |
|  |  |  |  | PLA |

**SUCRA**

| LEM10 | 94.1% |
| --- | --- |
| LEM5 | 81.9% |
| VOR10 | 70.0% |
| VOR5 | 73.0% |
| PLA | 7.2% |

**Global heterogeneity**

Between study variance (𝜏^2^): 0.000 (heterogeneity assessment: low)

**Random-effects design-by-treatment interaction model**

P value: 0.832

**Local heterogeneity (I^2^) and incoherence (SIDE test)**

|  | I^2^ (%) | P value (SIDE test) |
| --- | --- | --- |
| LEM10 vs LEM5 | 30.0 | No inconsistency |
| LEM10 vs PLA | 0.0 | No inconsistency |
| LEM5 vs PLA | 0.0 | No inconsistency |

**CINeMA confidence rating**

| Comparison | Number of studies | Within-study bias | Reporting bias | Indirectness | Imprecision | Heterogeneity | Incoherence | Confidence rating |
| --- | --- | --- | --- | --- | --- | --- | --- | --- |
| LEM10 vs LEM5 | 2 | No concerns | Some concerns | No concerns | No concerns | Major concerns | No concerns | Moderate |
| LEM10 vs PLA | 3 | No concerns | Some concerns | No concerns | No concerns | Major concerns | No concerns | Moderate |
| LEM5 vs PLA | 3 | No concerns | Some concerns | No concerns | No concerns | Major concerns | No concerns | Moderate |
| VOR10 vs VOR5 | 1 | No concerns | Some concerns | No concerns | Major concerns | No concerns | Major concerns | Low |
| VOR10 vs PLA | 1 | No concerns | Some concerns | No concerns | No concerns | Major concerns | Major concerns | Low |
| VOR5 vs PLA | 1 | No concerns | Some concerns | No concerns | No concerns | Major concerns | Major concerns | Low |
| LEM10 vs VOR10 | 0 | No concerns | Some concerns | No concerns | Major concerns | No concerns | Major concerns | Very low |
| LEM10 vs VOR5 | 0 | No concerns | Some concerns | No concerns | Major concerns | No concerns | Major concerns | Very low |
| LEM5 vs VOR10 | 0 | No concerns | Some concerns | No concerns | Major concerns | No concerns | Major concerns | Very low |
| LEM5 vs VOR5 | 0 | No concerns | Some concerns | No concerns | Major concerns | No concerns | Major concerns | Very low |

**Appendix S9. Adverse events–related discontinuation**

7 studies, 2798 participants

**League table (OR with 95% CI)**

| LEM10 | 2.232 (0.836, 5.963) | 3.332 (0.245, 45.345) | 3.361 (0.247, 45.728) | 1.647 (0.698, 3.888) |
| --- | --- | --- | --- | --- |
|  | LEM5 | 1.493 (0.102, 21.846) | 1.505 (0.103, 22.032) | 0.738 (0.256, 2.129) |
|  |  | VOR10 | 1.009 (0.062, 16.276) | 0.494 (0.042, 5.817) |
|  |  |  | VOR5 | 0.490 (0.042, 5.768) |
|  |  |  |  | PLA |

**SUCRA**

| LEM10 | 11.9% |
| --- | --- |
| LEM5 | 35.9% |
| VOR10 | 70.1% |
| VOR5 | 68.9% |
| PLA | 50.5% |

**Global heterogeneity**

Between study variance (𝜏^2^): 0.000 (heterogeneity assessment: low)

**Random-effects design-by-treatment interaction model**

P value: 0.998

**Local heterogeneity (I^2^) and incoherence (SIDE test)**

|  | I^2^ (%) | P value (SIDE test) |
| --- | --- | --- |
| LEM10 vs LEM5 | 0.0 | No inconsistency |
| LEM10 vs PLA | 0.0 | No inconsistency |
| LEM5 vs PLA | 0.0 | No inconsistency |
| VOR10 vs VOR5 | na | No inconsistency |
| VOR10 vs PLA | na | No inconsistency |
| VOR5 vs PLA | na | No inconsistency |

**CINeMA confidence rating**

| Comparison | Number of studies | Within-study bias | Reporting bias | Indirectness | Imprecision | Heterogeneity | Incoherence | Confidence rating |
| --- | --- | --- | --- | --- | --- | --- | --- | --- |
| LEM10 vs LEM5 | 4 | No concerns | Some concerns | No concerns | Major concerns | No concerns | No concerns | Moderate |
| LEM10 vs PLA | 4 | No concerns | Some concerns | No concerns | Major concerns | No concerns | No concerns | Moderate |
| LEM5 vs PLA | 4 | No concerns | Some concerns | No concerns | Major concerns | No concerns | No concerns | Moderate |
| VOR10 vs VOR5 | 2 | No concerns | Some concerns | No concerns | Major concerns | No concerns | Major concerns | Low |
| VOR10 vs PLA | 2 | No concerns | Some concerns | No concerns | No concerns | Major concerns | Major concerns | Low |
| VOR5 vs PLA | 2 | No concerns | Some concerns | No concerns | No concerns | Major concerns | Major concerns | Low |
| LEM10 vs VOR10 | 0 | No concerns | Some concerns | No concerns | Major concerns | No concerns | Major concerns | Very low |
| LEM10 vs VOR5 | 0 | No concerns | Some concerns | No concerns | Major concerns | No concerns | Major concerns | Very low |
| LEM5 vs VOR10 | 0 | No concerns | Some concerns | No concerns | Major concerns | No concerns | Major concerns | Very low |
| LEM5 vs VOR5 | 0 | No concerns | Some concerns | No concerns | Major concerns | No concerns | Major concerns | Very low |

**Appendix S10. At least one adverse event**

7 studies, 2798 participants

**League table (OR with 95% CI)**

| LEM10 | 1.277 (0.975, 1.672) | 1.579 (0.890, 2.803) | 1.779 (0.995, 3.181) | **2.232 (1.666, 2.991)** |
| --- | --- | --- | --- | --- |
|  | LEM5 | 1.237 (0.691, 2.214) | 1.394 (0.773, 2.512) | **1.748 (1.284, 2.380)** |
|  |  | VOR10 | 1.127 (0.702, 1.810) | 1.413 (0.863, 2.315) |
|  |  |  | VOR5 | 1.254 (0.759, 2.072) |
|  |  |  |  | PLA |

**SUCRA**

| LEM10 | 15.1% |
| --- | --- |
| LEM5 | 19.9% |
| VOR10 | 41.6% |
| VOR5 | 48.9% |
| PLA | 85.5% |

**Global heterogeneity**

Between study variance (𝜏^2^): 0.000 (heterogeneity assessment: low)

**Random-effects design-by-treatment interaction model**

P value: 0.881

**Local heterogeneity (I^2^) and incoherence (SIDE test)**

|  | I^2^ (%) | P value (SIDE test) |
| --- | --- | --- |
| LEM10 vs LEM5 | 0.0 | No inconsistency |
| LEM10 vs PLA | 0.0 | No inconsistency |
| LEM5 vs PLA | 0.0 | No inconsistency |
| VOR10 vs VOR5 | 0.0 | No inconsistency |
| VOR10 vs PLA | 0.0 | No inconsistency |
| VOR5 vs PLA | 0.0 | No inconsistency |

**CINeMA confidence rating**

| Comparison | Number of studies | Within-study bias | Reporting bias | Indirectness | Imprecision | Heterogeneity | Incoherence | Confidence rating |
| --- | --- | --- | --- | --- | --- | --- | --- | --- |
| LEM10 vs LEM5 | 4 | No concerns | Some concerns | No concerns | No concerns | No concerns | Major concerns | Moderate |
| LEM10 vs PLA | 4 | No concerns | Some concerns | No concerns | No concerns | No concerns | Major concerns | Moderate |
| LEM5 vs PLA | 4 | No concerns | Some concerns | No concerns | No concerns | No concerns | Major concerns | Moderate |
| VOR10 vs VOR5 | 2 | No concerns | Some concerns | No concerns | Major concerns | No concerns | Major concerns | Low |
| VOR10 vs PLA | 2 | No concerns | Some concerns | No concerns | No concerns | Major concerns | Major concerns | Low |
| VOR5 vs PLA | 2 | No concerns | Some concerns | No concerns | No concerns | Major concerns | Major concerns | Low |
| LEM10 vs VOR10 | 0 | No concerns | Some concerns | No concerns | Major concerns | No concerns | Major concerns | Very low |
| LEM10 vs VOR5 | 0 | No concerns | Some concerns | No concerns | Major concerns | No concerns | Major concerns | Very low |
| LEM5 vs VOR10 | 0 | No concerns | Some concerns | No concerns | Major concerns | No concerns | Major concerns | Very low |
| LEM5 vs VOR5 | 0 | No concerns | Some concerns | No concerns | Major concerns | No concerns | Major concerns | Very low |

**Appendix S11. Somnolence**

7 studies, 2798 participants

**League table (OR with 95% CI)**

| LEM10 | **1.682 (1.059, 2.672)** | 1.738 (0.438, 6.893) | 2.126 (0.525, 8.619) | **5.232 (2.470, 11.083)** |
| --- | --- | --- | --- | --- |
|  | LEM5 | 1.033 (0.256, 4.172) | 1.264 (0.306, 5.215) | **3.110 (1.421, 6.805)** |
|  |  | VOR10 | 1.224 (0.515, 2.908) | 3.011 (0.948, 9.562) |
|  |  |  | VOR5 | 2.461 (0.755, 8.019) |
|  |  |  |  | PLA |

**SUCRA**

| LEM10 | 13.1% |
| --- | --- |
| LEM5 | 25.0% |
| VOR10 | 42.2% |
| VOR5 | 46.2% |
| PLA | 91.9% |

**Global heterogeneity**

Between study variance (𝜏^2^): 0.000 (heterogeneity assessment: low)

**Random-effects design-by-treatment interaction model**

P value: 0.833

**Local heterogeneity (I^2^) and incoherence (SIDE test)**

|  | I^2^ (%) | P value (SIDE test) |
| --- | --- | --- |
| LEM10 vs LEM5 | 0.0 | No inconsistency |
| LEM10 vs PLA | 10.7 | No inconsistency |
| LEM5 vs PLA | 0.0 | No inconsistency |
| VOR10 vs VOR5 | 0.0 | No inconsistency |
| VOR10 vs PLA | 0.0 | No inconsistency |
| VOR5 vs PLA | 0.0 | No inconsistency |

**CINeMA confidence rating**

| Comparison | Number of studies | Within-study bias | Reporting bias | Indirectness | Imprecision | Heterogeneity | Incoherence | Confidence rating |
| --- | --- | --- | --- | --- | --- | --- | --- | --- |
| LEM10 vs LEM5 | 4 | No concerns | Some concerns | No concerns | No concerns | Major concerns | No concerns | Moderate |
| LEM10 vs PLA | 4 | No concerns | Some concerns | No concerns | No concerns | Major concerns | No concerns | Moderate |
| LEM5 vs PLA | 4 | No concerns | Some concerns | No concerns | No concerns | Major concerns | No concerns | Moderate |
| VOR10 vs VOR5 | 2 | No concerns | Some concerns | No concerns | Major concerns | No concerns | Major concerns | Low |
| VOR10 vs PLA | 2 | No concerns | Some concerns | No concerns | No concerns | Major concerns | Major concerns | Low |
| VOR5 vs PLA | 2 | No concerns | Some concerns | No concerns | No concerns | Major concerns | Major concerns | Low |
| LEM10 vs VOR10 | 0 | No concerns | Some concerns | No concerns | Major concerns | No concerns | Major concerns | Very low |
| LEM10 vs VOR5 | 0 | No concerns | Some concerns | No concerns | Major concerns | No concerns | Major concerns | Very low |
| LEM5 vs VOR10 | 0 | No concerns | Some concerns | No concerns | Major concerns | No concerns | Major concerns | Very low |
| LEM5 vs VOR5 | 0 | No concerns | Some concerns | No concerns | Major concerns | No concerns | Major concerns | Very low |

**Appendix S12. Headache**

7 studies, 2798 participants

**League table (OR with 95% CI)**

| LEM10 | 0.813 (0.463, 1.429) | 0.560 (0.099, 3.173) | 0.691 (0.127, 3.751) | 1.445 (0.754, 2.769) |
| --- | --- | --- | --- | --- |
|  | LEM5 | 0.689 (0.122, 3.872) | 0.850 (0.158, 4.577) | 1.776 (0.946, 3.337) |
|  |  | VOR10 | 1.234 (0.293, 5.190) | 2.580 (0.517, 12.875) |
|  |  |  | VOR5 | 2.090 (0.439, 9.957) |
|  |  |  |  | PLA |

**SUCRA**

| LEM10 | 50.9% |
| --- | --- |
| LEM5 | 45.1% |
| VOR10 | 29.7% |
| VOR5 | 36.4% |
| PLA | 70.1% |

**Global heterogeneity**

Between study variance (𝜏^2^): 0.000 (heterogeneity assessment: low)

**Random-effects design-by-treatment interaction model**

P value: 0.705

**Local heterogeneity (I^2^) and incoherence (SIDE test)**

|  | I^2^ (%) | P value (SIDE test) |
| --- | --- | --- |
| LEM10 vs LEM5 | 0.0 | No inconsistency |
| LEM10 vs PLA | 0.0 | No inconsistency |
| LEM5 vs PLA | 0.0 | No inconsistency |
| VOR10 vs VOR5 | 48.2 | No inconsistency |
| VOR10 vs PLA | 39.5 | No inconsistency |
| VOR5 vs PLA | 0.0 | No inconsistency |

**CINeMA confidence rating**

| Comparison | Number of studies | Within-study bias | Reporting bias | Indirectness | Imprecision | Heterogeneity | Incoherence | Confidence rating |
| --- | --- | --- | --- | --- | --- | --- | --- | --- |
| LEM10 vs LEM5 | 4 | No concerns | Some concerns | No concerns | Major concerns | No concerns | No concerns | Moderate |
| LEM10 vs PLA | 4 | No concerns | Some concerns | No concerns | Major concerns | No concerns | No concerns | Moderate |
| LEM5 vs PLA | 4 | No concerns | Some concerns | No concerns | Major concerns | No concerns | No concerns | Moderate |
| VOR10 vs VOR5 | 2 | No concerns | Some concerns | No concerns | Major concerns | No concerns | Major concerns | Low |
| VOR10 vs PLA | 2 | No concerns | Some concerns | No concerns | No concerns | Major concerns | Major concerns | Low |
| VOR5 vs PLA | 2 | No concerns | Some concerns | No concerns | No concerns | Major concerns | Major concerns | Low |
| LEM10 vs VOR10 | 0 | No concerns | Some concerns | No concerns | Major concerns | No concerns | Major concerns | Very low |
| LEM10 vs VOR5 | 0 | No concerns | Some concerns | No concerns | Major concerns | No concerns | Major concerns | Very low |
| LEM5 vs VOR10 | 0 | No concerns | Some concerns | No concerns | Major concerns | No concerns | Major concerns | Very low |
| LEM5 vs VOR5 | 0 | No concerns | Some concerns | No concerns | Major concerns | No concerns | Major concerns | Very low |

**Appendix S13. Individual adverse events that were very rare**

|  | Placebo | LEM10 | LEM5 | VOR10 | VOR5 |
| --- | --- | --- | --- | --- | --- |
| Death | 0.000% | 0.000% | 0.000% | 0.000% | 0.000% |
| Suicidal behavior | 0.000% | 0.000% | 0.000% | 0.000% | 0.000% |
| Suicidal ideation | 0.000% | 0.005% | 0.000% | 0.000% | 0.000% |
| Fatigue | 0.000% | 0.018% | 0.016% | 0.000% | 0.000% |
| Dizziness | 0.014% | 0.010% | 0.015% | 0.000% | 0.000% |
| Falls | 0.000% | 0.000% | 0.000% | 0.000% | 0.000% |
| Nightmares | 0.000% | 0.016% | 0.009% | 0.000% | 0.000% |
| Cataplexy | 0.000% | 0.000% | 0.000% | 0.000% | 0.000% |
| Sleep paralysis | 0.000% | 0.016% | 0.008% | 0.000% | 0.000% |

*Although our network meta-analysis included these outcomes, we also reported incidence rates of these adverse events, as clinicians and patients with insomnia are particularly concerned with their occurrence.
